# Factors associated with lipid control in outpatients with heart failure

**DOI:** 10.3389/fcvm.2023.1153310

**Published:** 2023-04-19

**Authors:** Anan S. Jarab, Walid Al-Qerem, Hanan Hamam, Shrouq Abu Heshmeh, Tareq L. Mukattash, Eman A. Alefishat

**Affiliations:** ^1^ Department of Clinical Pharmacy, Faculty of Pharmacy, Jordan University of Science and Technology, Irbid, Jordan; ^2^ College of Pharmacy, Al Ain University, Abu Dhabi, United Arab Emirates; ^3^ Department of Pharmacy, Faculty of Pharmacy, Al-Zaytoonah University of Jordan, Amman, Jordan; ^4^ Department of Pharmacology, College of Medicine and Health Science, Khalifa University of Science and Technology, Abu Dhabi, United Arab Emirates; ^5^ Department of Biopharmaceutics and Clinical Pharmacy, Faculty of Pharmacy, The University of Jordan, Amman, Jordan; ^6^ Center for Biotechnology, Khalifa University, Abu Dhabi, United Arab Emirates

**Keywords:** heart failure, cardiovascular disease, lipid control, factors, interventions

## Abstract

**Background:**

Dyslipidemia is common among patients with heart failure, and it negatively impacts clinical outcomes. Limited data regarding the factors associated with poor lipid control in patients with HF patients. Therefore, this study aimed to evaluate lipid control and to explore the factors associated with poor lipid control in patients with HF.

**Methods:**

The current cross-sectional study was conducted at outpatient cardiology clinics at two major hospitals in Jordan. Variables including socio-demographics, biomedical variables, in addition to disease and medication characteristics were collected using medical records and custom-designed questionnaire. Medication adherence was assessed using the validated 4-item Medication Adherence Scale. Binary logistic regression analysis was conducted to explore significant and independent predictors of poor lipid control among the study participants.

**Results:**

A total of 428 HF patients participated in the study. Results showed that 78% of the participants had poor lipid control. The predictors that were associated with poor lipid control included uncontrolled BP (OR = 0.552; 95% CI: 0.330–0.923; *P* < 0.05), higher Hb levels (OR = 1.178; 95% CI: 1.013–1.369; *P* < 0.05), and higher WBC (OR = 1.133; 95% CI: 1.031–1.246; *P* < 0.05).

**Conclusions:**

This study revealed poor lipid control among patients with HF. Future intervention programs should focus on blood pressure control in order to improve health outcomes among HF patients with dyslipidemia.

## Introduction

Heart failure (HF) is a complex clinical syndrome with signs and symptoms that are caused by any functional or structural impairment of ventricular filling or ejection of blood (1). Patients with HF suffer from significant physical, psychological, social burdens, and various life-limiting symptoms including dyspnea, fatigue, edema, sleeping difficulties, chest pain, depression (2–4). In 2018, it was estimated that 64.3 million patients were suffering from HF worldwide (5). Previous studies reported that the prevalence of HF is expected to rise by 46% from 2012 to 2030, resulting in more than 8 million people aged 18 years and older having the disease (6). Despite the existing HF management guidelines and the effective treatment, outcomes are still suboptimal, and the mortality and morbidity rates remained high in addition to the enormous economic burden (7, 8). Recent Jordanian statistics reported that cardiovascular diseases (CVD) including HF contributed to about 37% of all deaths in the country (9).

Dyslipidemia is a well-established modifiable risk factor for CVDs (10). Comorbidities such as hypertension, type 2 diabetes, obesity, dyslipidemia, and metabolic syndrome are common in patients with HF and negatively impact clinical outcomes (11, 12). Elevated low-density lipoprotein (LDL-C) cholesterol and decreased high-density lipoprotein cholesterol (HDL-C) have been shown to be associated with reduced cardiac function (13). A previous study reported that the inflammation associated with the pathogenesis of dyslipidemia plays a significant role in the exacerbation of HF (14). In 2011, the prevalence rate of dyslipidemia in the United States was estimated to be 57% (15), while much higher prevalence was found in a cross-sectional community-based study conducted in Jordan (76%) (16). Hypercholesterolemia in Jordan almost doubled from 23.0% in 1994 to 44.3% in 2017, and hypertriglyceridemia increased from 23.8% in 1994 to 41.9% in 2017 (17).

The negative impact of dyslipidemia on HF prognosis necessitates exploring the factors associated with poor lipid control in order to be addressed in future intervention programs which aim at improving health outcomes among patients with HF. Earlier studies reported different predictors of uncontrolled lipid profile in patients with dyslipidemia (18–20). To the best of our knowledge, the current study is the first one which investigates the factors associated with poor lipid control among HF patients, not only in Jordan, but even worldwide.

## Methods

### Study design and subjects

In this cross-sectional study, we interviewed 428 HF patients out of 550 who visited two outpatient cardiology clinics in King Abdullah University Hospital and Al Bashir Hospital in the period from August 2021 through April 2022. Eligible participants were HF patients aged 18 years and older, had HF for at least 6 months, were receiving at least one medication for heart failure management, and consented to participate in the study. Patients were excluded if they had an acute decompensation of HF or an active listing for heart transplantation or cognitive impairment. Patients who were eligible to participate were informed that participation is voluntary, they have the right to withdraw at any time, and their medical care and treatment will not be affected whether they participate or not. The participants were also informed that the collected data will only be used for the research purposes and will be kept at the Principal Investigator's office to ensure confidentiality. An informed consent was obtained from each patient agreed to participate in the study. Each interview with patients took approximately 10–15 min to be completed.

### Study instruments and data collection

During outpatient clinics visits, the researcher pharmacist HH collected participants' sociodemographic data using a custom-designed questionnaire, which included participants' age, gender, body mass index (BMI), marital status, place of residency, living arrangements, education, occupation status, monthly income, smoking status, physical activity, and family history of cardiovascular diseases. The cardiologists established the diagnosis of HF after utilizing the HF diagnostic tests including echocardiography and the elevated pro-NT-BNP. The medical records were used to collect information about medical history including duration of HF, NYHA heart failure classification, the presence and number of other comorbidities such as hypertension, diabetes, ischemic heart disease, chronic kidney disease, dyslipidemias, thyroid dysfunction. The collected information also included the total number of medications, number of heart failure medications, frequency of taking medications, medications side effects, fears of medications side effects, medications satisfaction, and receiving digoxin, anticoagulant and different medications for blood pressure and lipids control among patients with HF. The researcher also used the medical records to obtain biomedical data and laboratory tests including low-density lipoproteins (LDL), high-density lipoproteins (HDL), triglycerides (TGs), total cholesterol (TC), hemoglobin A1C, random blood glucose, systolic blood pressure (SBP), diastolic blood pressure (DBP), ejection fraction, serum creatinine, white blood cells, red blood cells and hemoglobin. According to the 2018 guideline on the management of blood cholesterol, the patients were considered to have uncontrolled lipid profile if they had any one of the following: LDL-C ≥100 mg/dl (≥70 mg/dl for very high risk patients), TG ≥150 mg/dl or HDL-C <50 mg/dl for females and <40 mg/dl for males (21). Based on the new ACC/AHA blood pressure (BP) guidelines, the target for BP control in patients with HF is <130/80, so the patients were considered to have uncontrolled BP if they had BP readings of ≥130/80 (22). Patients' willingness to take medications as prescribed was measured by using the 4-item Medication Adherence Scale. This simple, validated 4-question survey, illustrated the variety of reasons for medication omission: forgetting, carelessness, stopping when feeling better or worse (23). In scoring the questionnaire, one score was given for each “yes” response, and each “no” response was given a score of zero. The scores were ranging from 0 to 4. Adherence was divided into three groups: high for those scoring zero, medium for those scoring one or two, and low for those scoring three or four. The validated Arabic version was used to assess medication adherence among HF patients in the current study (24). The surveys were completely self-reported, and patients who faced difficulty-completing questionnaires, had the questionnaires read to them without giving any interpretation of the questions.

### Data analysis

Descriptive and analytical statistics were conducted utilizing the Statistics Package for the Social Sciences (SPSS version 26 from IBM). Continuous and categorical variables were described by means (standard deviations) and frequencies (percentages), respectively. The independent sample *t*-test and the Mann–Whitney *U*-test were applied for normally and non-normally distributed continuous variables. In order to investigate if there is an association between categorical variables and lipid control the Pearson Chi-square test was constructed and the variables with a *P* < 0.05 were fitted into the binary logistic regression to determine the significant and independent predictors of lipid control.

### Ethical approval

The study has been carried out in accordance with the Declaration of Helsinki (1975) for experiments involving human subjects. Ethical approval was obtained from the Institutional Review Board (IRB) of KAUH at Jordan University of Science and Technology (Ref. # 32/141/2021).

## Results

In total, 428 HF patients agreed to participate in the study. The participants' mean age was 63 ± 12 years. The majority of the participants were males (63.8%), married (94.4%), living with their families (94.6), living in the city (66.8%), did not complete their education (69.4%), unemployed or retired (77.6%), had a monthly income less than 500 JD (67.1%), non-smokers (69.9%), and were not physically active (88.1%). More than half of the patients had no family history of cardiovascular diseases (51.2%). Other demographic characteristics are presented in Table 1.

**Table 1:** Demographic characteristics of the study participants (*n* = 428).

Characteristic	Mean (±SD)	*n* (%)
Age (years)	63 (±12)	
Gender		
Male		273 (63.8)
Female		155 (36.2)
Body mass index (kg/m^2^)		
Normal (≤24.9)		90 (21.8)
Overweight (25–29.9)		161 (39)
Obese (≥30)		162 (39.2)
Marital status		
Married		404 (94.4)
Single/divorced/widowed		24 (5.6)
Living conditions		
Living alone		23 (5.4)
Living with family/other		405 (94.6)
Residency		
City		286 (66.8)
Countryside		142 (33.2)
Education		
University/collage		131 (30.6)
Less than collage		297 (69.4)
Working status		
Employed		96 (22.4)
Unemployed/retired		332 (77.6)
Monthly income		
Less than 500 JD		287 (67.1)
500–1,000 JD^2^		141 (32.9)
Smoking status		
Current smoker		129 (30.1)
Non-smoker		299 (69.9)
Physical activity		
Active		51 (11.9)
Not active		377 (88.1)
Family history of CVD		
Yes		209 (48.8)
No		219 (51.2)

SD, standard deviation; JD, Jordanian dinar; CVD, cardiovascular disease.

As shown in Table 2, the duration of HF in years had a mean of 6.41 (±6.16). The mean number of comorbid diseases was 3 (±1) and the most common comorbidities among the participants were hypertension (77.1%), type 2 diabetes (61.2%), and ischemic heart disease (IHD) (64.3%). The majority of the patients were classified as group III/IV according to NYHA classification (69.6%). The patients received 8 (±3) medications on average, and the mean number of HF medications was 3 (±1). The most prescribed HF medications were beta blockers (88.6%), loop diuretics (67.5%), and angiotensin-converting enzyme blockers/angiotensin-receptor blockers (66.8%). Most of the patients reported moderate adherence to HF medications (83.6%), 8.9% showed low adherence, while only 7.5% had high medication adherence. According to the 2018 Guidelines on the Management of Blood Cholesterol, 245 patients out of 428 (57.24%) were classified as very high-risk patients. Out of 303 patients who received statin therapy, 270 patients (89.1%) received high intensity statin and 33 patients (6.7%) received moderate intensity statin. Among the patients who received high intensity statin, atorvastatin 40/80 mg was prescribed for 93.3% and rosuvastatin 20/40 mg was prescribed for 6.7% of the participants. On the other hand, all the patients who received moderate intensity statin were prescribed atorvastatin 10/20 mg. The majority of the patients were satisfied with their medications (77.3%), had no fears toward medications' side effects (71.3%), and did not experience any side effects (68.0%). The means of TGs, TC, LDL, and HDL were 1.96 (±1.169), 4.18 (±1.288), 2.47 (±1.12), and 0.993 (±0.316) mmol/L, respectively. Other laboratory tests results are presented in Table 3.

**Table 2:** Disease and medication characteristics of the study participants (*n* = 428).

Variables	*n* (%)	Mean (SD)
Disease related		
Duration of heart failure (years)		6.41 (±6.16)
Number of other chronic diseases		3 (±1)
NYHA classification		
I/II	130 (30.4)	
III/IV	298 (69.6)	
Type of comorbidities		
Hypertension	330 (77.1)	
Diabetes mellitus	262 (61.2)	
Ischemic heart diseases	275 (64.3)	
Chronic kidney disease	56 (13.1)	
Thyroid dysfunction	36 (8.4)	
Heart failure medications		
Aldosterone antagonist	81 (18.9)	
Digoxin	46 (10.7)	
ACEIs/ARBs	286 (66.8)	
Beta blocker	379 (88.6)	
Loop diuretic	289 (67.5)	
Vasodilator	79 (18.5)	
Calcium channel blocker	81 (18.9)	
Ivabradine	20 (4.7)	
Valsartan/sacubitril	31 (7.2)	
Other medications		
Anticoagulant	148 (34.6)	
Statin	303 (70.8)	
Number of medications		8 (±3)
Number of heart failure medications		3 (±1)
Medications frequency		
Once	78 (18.2)	
Twice	249 (58.2)	
Thrice or more	101 (23.6)	
Fear of medications’ side effects		
No	305 (71.3)	
Yes	123 (28.7)	
Medications satisfaction		
No	97 (22.7)	
Yes	331 (77.3)	
Experiencing side effects		
No	291 (68)	
Yes	137 (32)	

SD, standard deviation; NYHA, The New York Heart Association Classification; ACEI, angiotensin-converting enzyme inhibitor; ARB, angiotensin-receptor blocker.

**Table 3:** Laboratory tests of the study participants (*n* = 428).

Variable	Mean (±SD)
HbA1c (%)	7.28 (±1.97)
TGs (mmol/L)	1.96 (±1.169)
TC (mmol/L)	4.18 (±1.288)
LDL (mmol/L)	2.47 (±1.12)
HDL (mmol/L)	0.993 (±0.316)
Non-HDL Cholesterol (mmol/L)	2.94 (±1.47)
SCr (micmol/L)	108.15 (±78.43)
Hb (g/dl)	12.95 (±2.23)
WBCs count (*10^9^/L)	8.62 (±2.89)
RBCs count (*10^9^/L)	4.68 (±0.92)
EF (%)	42 (±10)
SBP (mmHg)	129 (±22)
DBP (mmHg)	76 (±12)

SD, standard deviation; Hb, hemoglobin; TGs, triglycerides; TC, total cholesterol; LDL, low-density lipoproteins; HDL, high-density lipoproteins, SCr, serum creatinine; WBCs, white blood cells; RBCs, red blood cells; EF, ejection fraction; SBP, systolic blood pressure; DBP, diastolic blood pressure.

Results of the univariate analysis showed that monthly income, hypertension control, LDL level, Hb level, WBCs count, and RBCs count were significantly associated with lipid control. Variables that were significantly associated with lipid control in the univariate analysis (<0.05) were included in the binary logistic regression model. As shown in Table 4, patients who had controlled hypertension had lower odds to be in the uncontrolled lipid group when compared to those who had uncontrolled hypertension (OR = 0.552; 95% CI: 0.330–0.923; *P* < 0.05). Higher Hb levels (OR = 1.178; 95% CI: 1.013–1.369; *P* < 0.05) and higher WBCs count (OR = 1.133; 95% CI: 1.031–1.246; *P* < 0.05) increased the odds to be in the uncontrolled lipid group.

**Table 4:** Multivariate analysis of the variables associated with lipid control.

Variable	OR (95% CI)	*P*
Hypertension control status		
Uncontrolled	Reference	0.024*
Controlled	0.552 (0.33–0.923)	
Haemoglobin level	1.178 (1.013–1.369)	0.033*
White blood cells count	1.133 (1.031–1.246)	0.01*

* Significant at *P* < 0.05.

## Discussion

Dyslipidemia is a common disorder and an important risk factor for coronary heart disease and stroke (10). Statins are so far the most effective cholesterol-lowering agents that are usually prescribed for patients who have or at high risk for cardiovascular diseases (25, 26). However, many patients are still having uncontrolled lipid status despite the use of statins or other dyslipidemia medications. Therefore, the aim of the current study was to explore the variables associated with poor lipid control in patients with HF.

Lipid control status of the current study patients was far below the expectations, with the majority of them showing uncontrolled lipid status (78.2%). Several previous studies have found inadequate lipid control in patients with different chronic diseases. A study conducted in Spain reported that poor low-density lipoprotein (LDL) cholesterol control was recognized in nearly 60% of the patients with a cardiovascular disease (18). Another study conducted in Ghana among patients with type 2 diabetes found that more than one third of the participating patients had high total cholesterol and LDL levels (27). Suboptimal lipid control was also recognized in patients with premature coronary artery disease (CAD) participated in a Mexican study using two different criteria for lipid control assessment (28). Additionally, poor lipid control was found in patients with CHD enrolled in studies conducted in India (29) and Spain (30). The disappointing rates of poor lipid control among patients with HF and other chronic diseases warrants exploring the factors that impede the achievement of optimal lipid control, and subsequently, develop advanced strategies that would overcome these obstacles and reach this goal.

Patients with uncontrolled BP in the current study showed significantly poorer lipid control than those with controlled BP. In comparison, a study conducted in Spain reported that the absence of antihypertensive treatment was a significant determinant of poor lipid control in patients with cardiovascular disease participated in the study (18). Another study of over 15 thousand population reported that the total and non-high-density lipoprotein (HDL) cholesterol levels significantly increased with the increase in systolic or diastolic blood pressure (31). Furthermore, a case-control study conducted in Iraq found that patients with hypertension had significantly higher total cholesterol, triglycerides, LDL cholesterol, and lower HDL cholesterol levels than the healthy volunteers (32). Hypertension and hyperlipidemia are closely interrelated, and the prevalence of hyperlipidemia in hypertensive patients was found to be higher than that of normotensive individuals (33). Additionally, a study conducted among patients with combined hypertension and hypercholesterolemia in the United States reported that only 9% of the participants had overall control of both conditions (34). The true association between high blood pressure and lipid profile is poorly understood, thus, further research in this area is warranted to investigate this relationship and uncover the true etiology behind this association, which would help guide the development of future BP management and lipid control programs, and eventually, improve controlling both BP and lipid status and optimize patients' health outcomes.

Hemoglobin (Hb) is a protein molecule that is found in red blood cells and is involved in carrying oxygen from the lungs to the body's tissues and returns carbon dioxide from the tissues back to the lungs (35). The current study found an association between higher hemoglobin concentration and poor lipid control. Comparable results were reported in a Mexican study conducted among patients with fatty liver disease, where patients who had high Hb concentration showed alterations in lipid profile (36). Besides, a study conducted in Finland found that higher hemoglobin levels were associated with unfavorable lipoprotein particles' size and increasing amount of both larger VLDL and smaller LDL particles, which increase the risk for cardiovascular diseases (37). On the other hand, an Iranian study investigated the relationship between anemia and lipid profile among the elderly population reported that the amount of lipids such as triglycerides and cholesterol were less in the anemic individuals compared with the non-anemic ones (38). Consistent results were also found in another study conducted among anemic Korean girls (39). These findings suggest that hemoglobin level affects lipid profile in a positive relationship, but the exact mechanism of such association is not well-established, which highlights the need for further research to fully understand the possible mechanisms and the effect of higher hemoglobin levels on lipid profile in order to avoid the negative consequences associated with uncontrolled lipid levels on patients' health.

The current study results showed a significant relationship between white blood count (WBC) and poor lipid profile. A study conducted among 2,873 participants found a significant relationship between white blood cells concentration and plasma lipid levels (40). A cross-sectional and longitudinal analysis of three large cohort studies reported a significant correlation between dyslipidemia and leukocytosis (41). Another study which analyzed baseline data of over 12 thousand men in the Multiple Risk Factor Intervention Trial reported that elevated leucocyte count was independently associated with atherogenic lipid profile, which is an important risk factor for CHD (42). These findings suggest that patients with higher WBCs concentration were more likely to have dyslipidemia. Besides, high lipid concentration increases the risk for cardiovascular diseases, which would further worsen the health status of HF patients, therefore, future disease management programs should put into consideration the variables that hinder reaching an optimal lipid control state in order to improve HF patients' health outcomes and prevent the negative consequences of poorly controlled lipid profile on their health.

The current study has some limitations, the self-report method used in this study may have affected the accuracy of patients' responses due to social desirability bias. However, this study used a sample size higher than the required which provided a robust findings and unique insights into the predictors of poor lipid control status among Jordanian patients with HF. The dietary pattern and its effect on lipid control was not investigated in this study. Furthermore, dyslipidemia in the present study could be attributed to the lack of physical activity, which was reported by the majority of the study participants, not receiving statin by some patients who were eligible to statin therapy, and the effect of statin dosage regimen-related factors. However, the effect of these factors on lipid control was not evaluated in the present study.

Overall, findings of the current study can provide valuable insights that can help health care providers and policymakers improve future HF management strategies and develop targeted interventions that address the challenges that prevent achieving an optimal lipid control among patients with HF. Such interventions should focus on improving BP control *via* enhancing lifestyle modifications, optimizing blood pressure control medications along with regular patient follow-up. In terms of future research, Hb and WBCs levels should be more involved to help understand the way they affect lipid control and develop appropriate strategies accordingly. Furthermore, the impact of dyslipidemia on morbidity and mortality among patients with HF is deemed necessary in future studies.

## Conclusion

The current study showed inadequate lipid control in patients with HF. Uncontrolled BP, higher levels of Hb, and higher WBC were significantly and independently associated with poor lipid control in HF patients. Future intervention programs should implement effective strategies that would improve BP control in patients with HF in order to reduce its negative impact on lipid control and other health outcomes among patients with HF.

## Data Availability

The original contributions presented in the study are included in the article, further inquiries can be directed to the corresponding author.
